# MiR-21 over-expression and Programmed Cell Death 4 down-regulation features malignant pleural mesothelioma

**DOI:** 10.18632/oncotarget.24644

**Published:** 2018-04-03

**Authors:** Lorenzo Nicolè, Rocco Cappellesso, Tiziana Sanavia, Vincenza Guzzardo, Ambrogio Fassina

**Affiliations:** ^1^ Department of Medicine, Surgical Pathology & Cytopathology Unit, University of Padova, Padova, Italy; ^2^ Department of Biomedical Informatics, Harvard Medical School, Boston, MA, USA

**Keywords:** PDCD4, miR-21, malignant pleural mesothelioma, immunohistochemistry, prognosis

## Abstract

**Background:**

Differential diagnosis between malignant pleural mesothelioma (MPM) and benign mesothelial conditions is still challenging and there is a lack of useful markers. Programmed cell death 4 (PDCD4) is a well-known tumor suppressor gene in several cancers, its post-transcriptional activity is directly controlled by miR-21, whose over-expression has been recently reported in MPM compared to normal mesothelium. Aim of this study was to test this suppressor gene as a possible new marker of malignant transformation in mesothelial cells, as well as a new prognostic marker.

**Methods:**

PDCD4 nuclear expression was assessed by immunohistochemistry (IHC) in 40 non-neoplastic pleural (NNP) and 40 MPM formalin-fixed and paraffin-embedded specimens. PDCD4 and miR-21 expressions were analyzed by qRT-PCR in all cases. *In situ* hybridization (ISH) of miR-21 was performed in 5 representative cases of both groups. The prognostic relevance of PDCD4 was assessed in a public available gene expression dataset.

**Results:**

IHC showed that PDCD4 nuclear expression was significantly lower in MPM than in NNP. PDCD4 was down-regulated, whereas miR-21 was over-expressed in MPM cases compared to NNP ones. ISH detected miR-21 only in MPM specimens. Down-expression of PDCD4 was found significantly associated with short overall survival in publicly available data.

**Conclusions:**

These findings highlighted a switch between PDCD4 and miR-21 expression in MPM. Further studies should assess the diagnostic reliability of these two markers for MPM in biopsy and effusion specimens.

## INTRODUCTION

Malignant pleural mesothelioma (MPM) is the most lethal tumor arising from the mesothelial cells that line the serosal cavities and it is strongly associated to the long-term inhalation of asbestos fibers. Although infrequent, MPM global incidence is rising dramatically and it is expected to peak in the next decade, in relation with the long latency time (approximately 40 years) [1].

According to the World Health Organization (WHO), there are three main MPM histotypes. The epithelioid type is the most common and it is mainly characterized by epithelial-shaped cells expressing epithelial markers such as cytokeratin 5/6; the sarcomatoid type is composed by spindle cells which express mesenchymal markers as vimentin and S100A4; the biphasic type is composed by both epithelial-shaped and spindle cells [2]. Typically, MPM is unresponsive to conventional therapies and the mean overall survival ranges only between 9 and 12 months [3]. Molecular pathways implicated in MPM are still largely unknown, but it is clear that more than a single alteration triggers tumorigenesis and progression. Asbestos fibers may trigger the transformation of mesothelial cells through the generation of reactive oxygen species, inducing a genomic damage and causing the release of different pro-inflammatory and carcinogenetic cytokines by the mesothelial and inflammatory cells (e.g. tumor necrosis factor-α, several interleukins, transforming growth factor-β, and platelet-derived growth factor) [4]. All these mechanisms are linked to the inactivation of oncosuppressor genes, such as the complex cyclin-dependent kinase inhibitor 2A (CDKN2A)/alternative reading frame (ARF), retinoblastoma, and p53 [5]. Moreover, mutations of NF2 gene have been reported in MPM; NF2 encodes for the protein merlin, which is a negative regulator of the mTOR-related pathway [4, 5]. Recently, germline mutations of BRCA1-associated protein (BAP1, another oncosuppressor gene encoding for a nuclear ubiquitin C-terminal hydrolase regulating the gene expression) have been linked to a form of familiar MPM and loss of BAP1 has been proposed as a promising marker of malignancy in mesothelial cells [6, 7]. Epigenetic alterations have also been described in MPM, including promoter methylation of oncosuppressor genes such as E-cadherin, fragile histidine triad, retinoic acid receptor-β, and WNT inhibitor factor 1 [8]. miRNA dysregulation has also been found in MPM [9], possibly involved in carcinogenesis [10]. In particular, several papers reported miR-21 over-expression in MPM [11–13] and we recently demonstrated its reliability as MPM marker [14]. However, miRNA quantification is not easily available in all laboratories and it would be preferable to use the more common immuno-cyto-chemistry. MiR-21 has been shown to specifically regulate Programmed Cell Death 4 (PDCD4) expression at post-transcriptional level [15], but no studies have addressed PDCD4 expression in MPM yet. PDCD4 is an oncosuppressor gene whose expression is frequently altered in cancer, causing the disruption of the apoptotic machinery. PDCD4 plays its function at both mRNA transcriptional and translational level. Specifically, PDCD4 inhibits the translation of several oncoproteins by suppressing the activity of eukaryotic initiation factors 4A and 4G (eIF4A, eIF4G) and affects gene transcription by interacting with the JNK/c-Jun/AP-1 pathway. The genes mainly regulated by the PDCD4 activity are p21, Cdk4, ornithine decarboxylase and carbonic anhydrase II16. The nuclear PDCD4 expression decrease during carcinogenesis can be considered as a possible indicator of malignant transformation [16, 17] and a suitable immunohistochemical marker to distinguish MPM from benign mesothelial conditions [13, 14].

Aim of this study is the evaluation of PDCD4 and miR-21 expressions both at RNA and protein levels in two cohorts of MPM and non-neoplastic pleural (NNP) samples. Moreover, the prognostic relevance of PDCD4 was assessed in a publicly available MPM gene expression dataset, testing for associations between PDCD4 and overall survival.

## RESULTS

**Figure 1:**
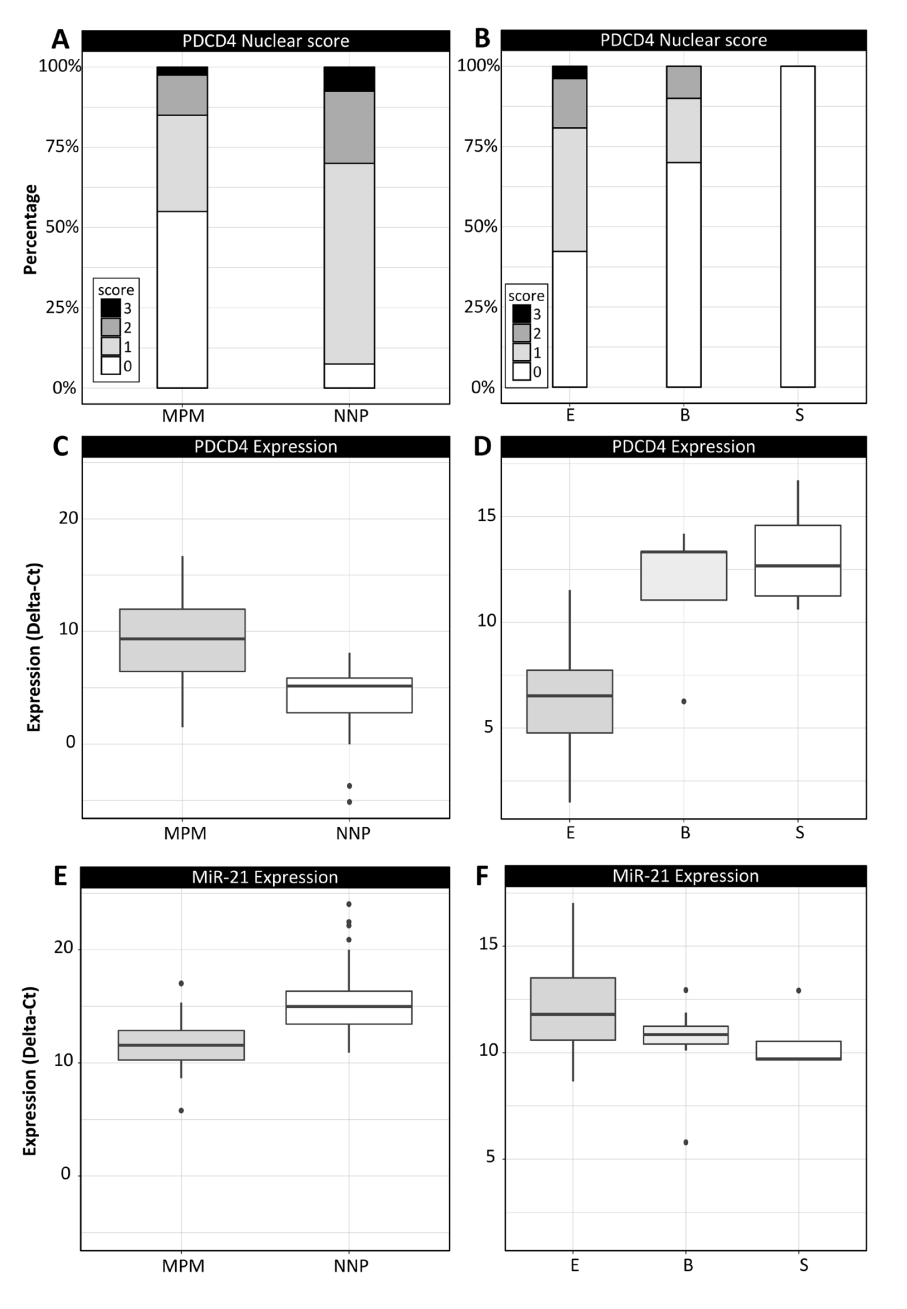
Immunohistochemical and qRT-PCR data analysis in MPM and NNP cohorts **(A-B)** distribution of IHC-based nuclear scores of PDCD4 in the two cohorts (A) and in the MPM histological subtypes (B); **(C-D)** PDCD4 expression in terms of ΔCt values in the two cohorts (C) and in the MPM histological subtypes (D); **(E-F)** miR-21 expression in terms of ΔCt values in the two cohorts (E) and in the MPM histological subtypes (F). Legend for the MPM subtypes: E: epithelioid, B: biphasic, S: sarcomatoid.

In MPM specimens, nuclear PDCD4 positive immunostaining was scored 0 in 22 cases, 1 in 12 cases, and 2 in 5 cases, 3 in one case; significantly lower with respect to NNP specimens (*p*=8.64e-05), where nuclear PDCD4 positive immunostaining was scored 0 in 3 cases, 1 in 25 cases, 2 in 9 cases and 3 in 3 cases (Figure 1A). Focusing on MPM histological subtypes, Mann–Whitney Wilcoxon test highlighted significantly higher scores for epithelioid subtype with respect to the joint group of biphasic and sarcomatoid samples (*p*=0.037; Figure 1B). There are no statistically significant differences between samples of normal pleura and samples of pleuritis (p=0.54), whereas the comparison between cases of pleuritis and mesotheliomas provides a significant p-value (p=0.05).

**Figure 2:**
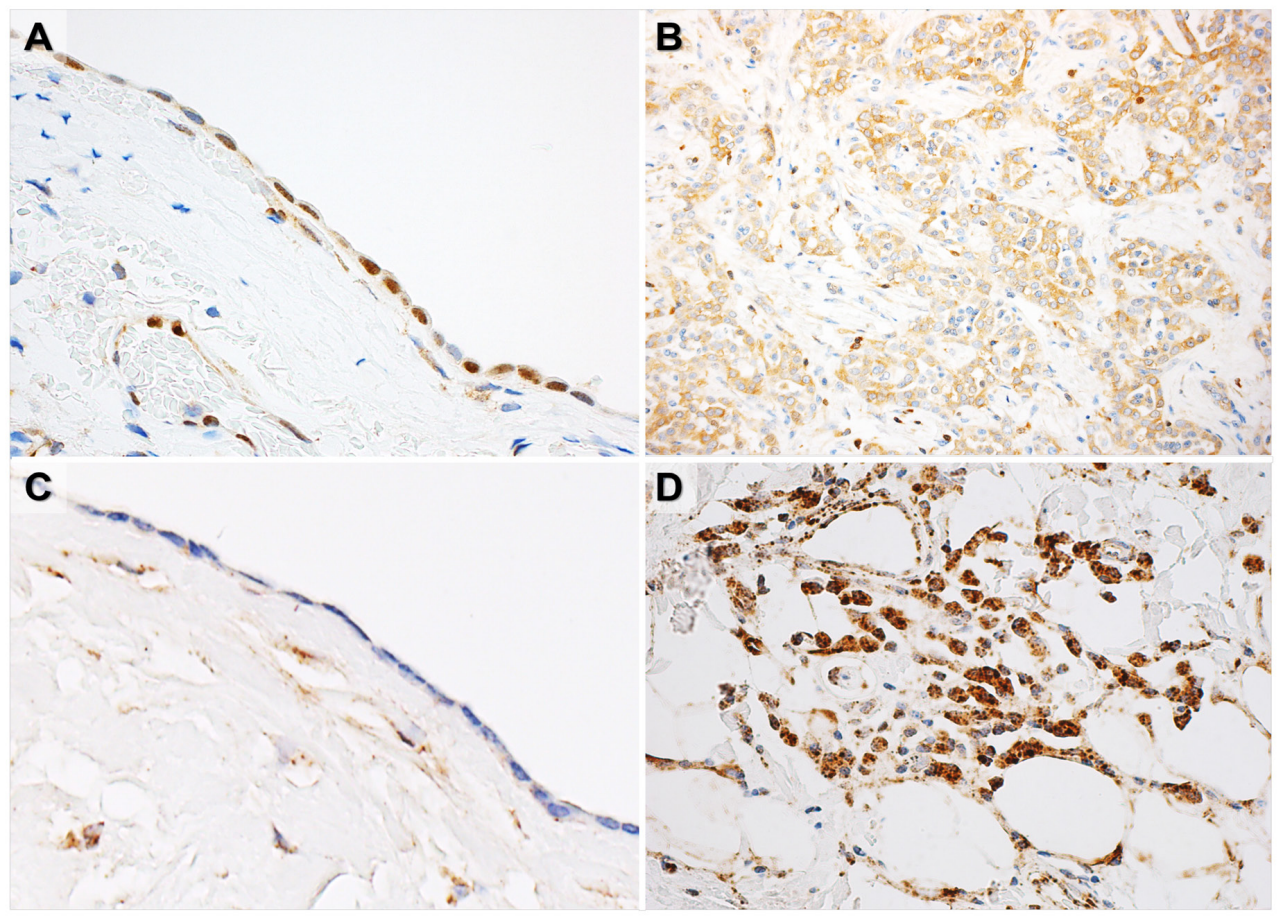
Representative figures of PDCD4 immunohistochemistry and *in situ* hybridization analysis of miR-21 **(A-C)** Normal mesothelial cells showed positive immunostain for nuclear PDCD4 (A) and undetectable level of miR-21 (C). **(B-D)** Malignant mesothelioma samples showed negative/low immunostain for PDCD4 (B) and high level of miR-21 (D).

PDCD4 and miR-21 expressions were investigated in MPM and NNP specimens also by qRT-PCR analysis (Figure 1C-1F, results reported in terms of ΔCt values). PDCD4 expression was significantly lower in MPM than NNP specimens (*p*=0.0003, Figure 1C), with significantly higher expression in epithelioid MPMs with respect to biphasic and sarcomatoid cases (*p*=0.003, Figure 1D). Conversely, miR-21 was found over-expressed in MPM samples compared to NNP ones (*p*=1.03e-08, Figure 1E), with lower expression in epithelioid cases compared to the other histopathological subtypes (*p*=0.033, Figure 1F). *In situ* hybridization analysis detected miR-21 in MPM specimens, while all the NNP samples analyzed were negative (Figure 2).

**Figure 3:**
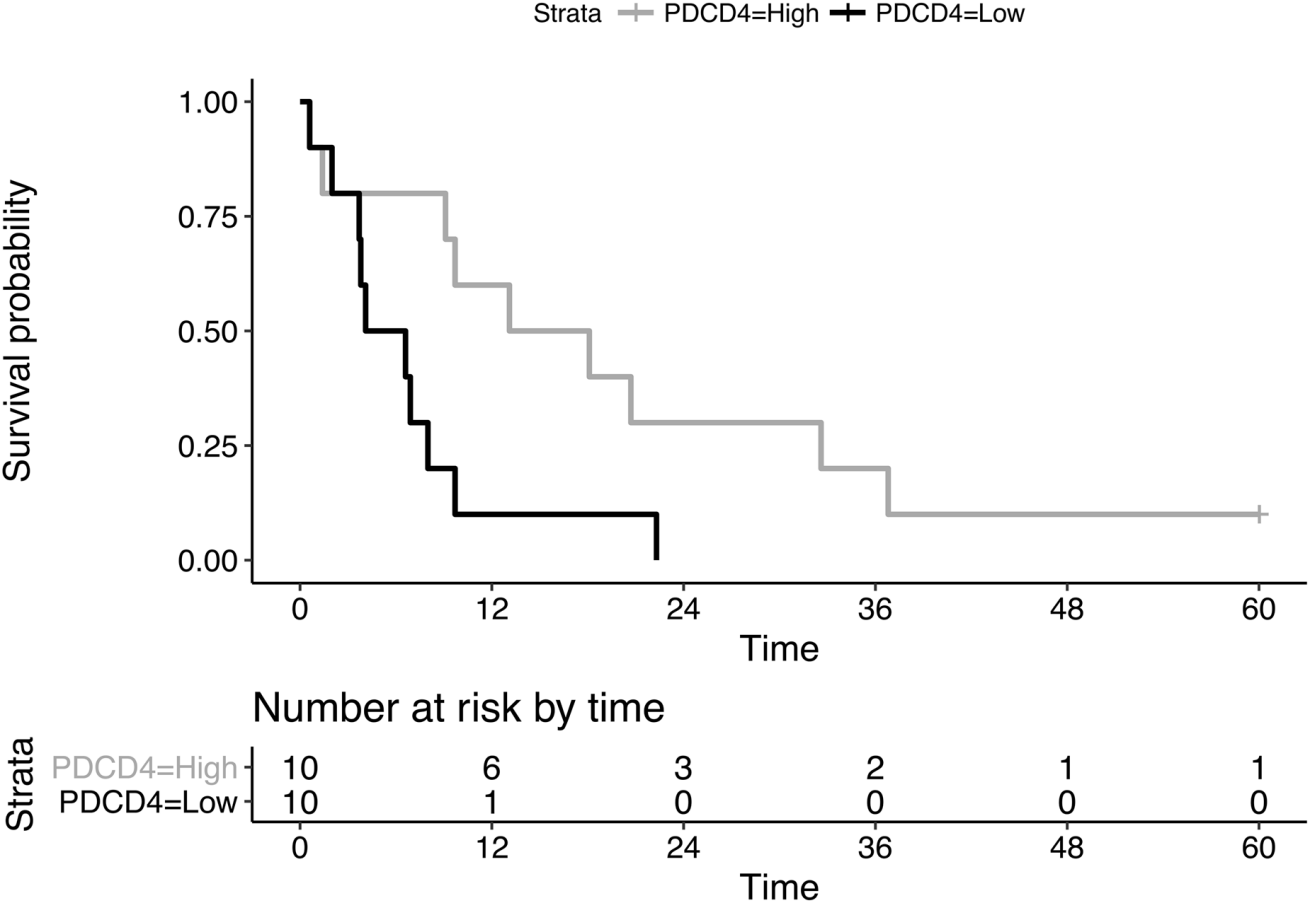
Kaplan-Meier curves of overall survival between PDCD4 expression-based subgroups Survival analysis performed on published MPM microarray data divided according to high (gray curve) and low (black curve) PDCD4 expression values.

Finally, PDCD4 survival analysis performed on the available public dataset [18] revealed a significant association between low expression levels of PDCD4 and short-term overall survival (*p*=0.024 Log-rank test, Figure 3). The survival model was not altered by the inclusion of confounding factors as gender, age and exposure to asbestos, which showed coefficients that were not statistically significant (*p*>0.4).

## DISCUSSION

MPM is one of the most lethal malignancies worldwide. Available therapies are mostly ineffective and both histological and cytological diagnoses remain a challenging task for pathologists [19]. Indeed, the strongest criterion to detect malignancy is the presence of stromal invasion within the context of aberrant mesothelial cells proliferation, whereas typically benign mesothelial proliferation associated with organizing pleuritis lacks the invasion by the mesothelial component [2]. However, in some cases cytological and morphological features of benign conditions may resemble those of MPM since glands or other type of cells may result incorporated into the thickened pleura, mimicking invasion [2, 20]. Therefore, in many cases these features alone are not always sufficient to achieve a definitive diagnosis and supportive ancillary tests would be necessary. The International Mesothelioma Interest Group recommended the use of at least four immunohistochemical markers (two in favor of and two against MPM diagnosis), however, diagnostic approaches to differentiate malignant versus benign mesothelial lesions using known markers (e.g. desmin, p53, IMP3, GLUT-1, CD146, and CD147) have proven to be inadequate [2]. Most recently, BAP1 immunostaining has been introduced demonstrating a diagnostic specificity of 100%, but sensitivity is still low since it does not exceed 60% [21]. Fluorescence *in situ* hybridization (FISH) for 9p21 has also been performed to this purpose, but the reported sensitivity and specificity for this technique are 61% and 100%, respectively [22]. Moreover, FISH is not yet available in all histo/cytopathological laboratories, it is time consuming, expensive, and it requires skilled dedicated personnel. These limitations may determine unacceptable delay in diagnosis as well as false negatives with relevant socio-economics and legal implications. miRNA quantification-based tests as well as metabonomic analysis by proton nuclear magnetic resonance have been proposed as an effective alternative to solve this differential diagnosis [13, 14, 23, 24]. However, despite the encouraging results, the limited accessibility to these approaches remarkably limits their application in the routine diagnostics. Therefore, the identification of new markers for MPM diagnosis remains a priority.

PDCD4 is a recently discovered onco-suppressor gene located at chromosome 10q24 that plays a crucial role in pro-apoptotic pathways and its impairment determines defective apoptosis. In the nucleus, PDCD4 interacts with the translation initiation factors eIF4A and eIF4G and suppresses oncogenic pathways such as cell transformation, tumorigenesis and invasion, regulating several downstream effectors as p21, Cdk4, ornithine decarboxylase, carbonic anhydrase II, and JNK/c-Jun/AP-1 [16]. PDCD4 loss or down-regulation has been correlated with tumor progression and poor prognosis in different tumors of thyroid, colon, esophagus, and ovary [25]. Moreover, studies on cell lines transfected with PDCD4 have shown its ability in inhibiting malignant behavior by the enhancement of apoptosis and chemo-sensitivity, suggesting this gene as a potential regulatory marker for novel therapeutic strategies [26].

The main post-transcriptional regulator of PDCD4 is represented by miR-21, an oncogenic microRNA that recognizes and directly binds the 3’-untraslated region of the PDCD4 transcript, blocking its translation into the protein [27]. MiR-21 over-expression has been reported in several cancers and, in some cases, this has been related to concurrent PDCD4 down-regulation [28–30]. miR-21 over-expression in MPM has been described in several studies, but its correlation with PDCD4 expression has not been investigated yet. Our findings highlighted a reduction of nuclear PDCD4 immunostaining and a substantial switch between PDCD4 and miR-21 expression levels in MPM compared to NNP samples. This is in agreement with the available evidence for other tumors [28]. Moreover, PDCD4 expression in MPM showed a trend from epithelioid to biphasic and sarcomatoid subtypes, suggesting a role in tumor progression and, more specifically, in epithelial-mesenchymal transition (EMT). This has already been demonstrated in gastric, colorectal, lung, and breast cancer cell lines and it seems to be promoted and sustained by miR-21 over-expression [31–34]. In particular, loss of PDCD4 has been associated with increased Snail/Slug expression, two markers of the EMT process [31]. EMT in MPM was linked to a worse prognosis and our analysis performed on a publicly available microarray gene expression dataset showed the impact of PDCD4 down-regulation in shortening the overall survival [35].

There are some major limits of this study. Firstly, the relative small size of the series of samples in NNP and MPM cohorts limits the evaluation of PDCD4 as a marker of malignant transformation in mesothelial cells, although the observed incidence of MPM in our area is among the highest in the western countries. In particular, the two cohorts include few cases of pleuritis and sarcomatoid mesotheliomas. For our analyses, samples of normal pleura and pleuritis, as well as samples from biphasic and sarcomatoid mesotheliomas, were considered together since no statistically significant changes were observed in these pairs of sample groups in terms of both IHC scores and RT-PCR expression of PDCD4 (*p*>0.3). However, as highlighted above, in the MPM cohort Figures 1B, 1D and 1F showed a trend across the three subtypes and we cannot exclude that, by increasing the number of both biphasic and sarcomatoid MPM cases, significant differences in PDCD4 expression may occur. Secondly, our study does not provide functional tests on mesothelial and MPM cell lines to directly demonstrate the effect of miR-21 expression on PDCD4 and on cell behavior, which has to be necessarily performed in further studies considering also the downstream effectors of PDCD4. Nevertheless, these analyses have already been accomplished in other tumors, thus the link between the inverse expression of miR-21 and PDCD4 can be reasonably assumed also in MPM [30]. Finally, the public dataset considered for the survival analysis includes only epithelioid MPM cases, therefore we were not able to assess differences of the overall survival on the other histotypes, which are expected to be even more significant between different levels of PDCD4 expression. An extensive search of possible datasets on MPM in the major databases for gene expression data such as GEO (Gene Expression Omnibus) and ArrayExpress showed that there are no datasets reporting all the three subtypes with available survival information on the patients. This aspect highlights the need of molecular data on large MPM cohorts across different subtypes to better assess novel diagnostic and prognostic markers.

## MATERIALS AND METHODS

### Histologic samples

From the archives of the Surgical Pathology and Cytopathology Unit of the University of Padova, formalin-fixed and paraffin-embedded (FFPE) biopsies of 40 NNP (8 pleuritis and 32 normal pleura) and 40 MPM (26 epithelioid, 4 sarcomatoid, and 10 biphasic) were collected. All NNP and MPM cases derived from patients that underwent surgical resection of malignancy or pleura biopsy from 2010 to 2014. Moreover, these cases were not included in our previous studies. Clinical and pathological data are reported in Table 1. All cases were reviewed and the diagnoses were confirmed by 3 pathologists (AF, RC, LN) according to the WHO classification. This study was approved by the Institutional Ethical Review Board of Padova University and we followed the Institute’s ethical regulations of research on human tissues.

**Table 1:** Clinical and pathological data of MPM and NNP patients

	N°	mean age ± SD	M/F
**NNP**			
Pleuritis	8	53,45 ± 3,56	4/5
Normal Pleura	32	41,15 ± 5,93	23/8
Total	40		27/13
**MPM**			
Epithelial	26	76,17 ± 4,23	18/8
Mixed	10	74,17 ± 4,51	7/3
Sarcomatoid	4	74,29 ± 6,11	3/1
Total	40		37/17

### Immunohistochemistry

Immunohistochemistry was performed on 4-μm to 5-μm thick FFPE sections from each tissue sample. Staining was automatically performed using a fully automated system (Bond™-maX; Leica, Newcastle Upon Tyne, UK). Sections were pre-treated using heat-mediated antigen retrieval with sodium citrate buffer (pH6, Epitope Retrieval Solution 1, Leica) for 30 minutes at 99°C. Specimens were then incubated with rabbit polyclonal anti-PDCD4 (catalog No. HPA001032; Atlas Antibodies, Stockholm, Sweden; 1:200) and detected using the Bond Polymer Refine Detection Kit (Leica) according to the manufacturer’s protocols. The staining was visualized with 3,3’-diaminobenzidine (DAB) and the slides were lightly counterstained with hematoxylin. Sections were then dehydrated, cleared, and mounted. We used samples from FFPE human tonsil tissue as positive controls and serum without the primary antibody as negative control. PDCD4 nuclear expression was jointly scored by 2 pathologists (LN and RC) in a whole section for each case, unaware of any clinical information. A semi-quantitatively 4-tiered scale based on the percentage of positive cells was used, with 0 indicating no stain, 1 indicating 1% to 30% staining, 2 indicating 31% to 70% staining, and 3 indicating 71% to 100% staining.

### *In situ* hybridization

Reactions were performed on 4-μm to 5-μm thick FFPE sections from 5 NNP and 5 MPM cases randomly selected, using the GenPoint Catalyzed Signal Amplification System (DakoCytomation, Glostrup, Denmark) according to the manufacturer’s protocol. 5’-biotin-labeled miR-21 miRCURY LNA microRNA detection probe (Exiqon, Vedbaek, Denmark) and the scrambled negative control probe (U6; Exiqon) at a final concentration of 200 nM were applied. The slides were finally counterstained with hematoxylin. Reactions were jointly assessed by 2 pathologists (LN and RC) and they were considered positive when granular cytoplasmic staining was present.

### RNA extraction

NNP and MPM samples were enriched in the neoplastic component by manual microdissection. Briefly, 5 consecutive, unstained, 10-μm thick FFPE sections of each specimen were scraped in a 1.5 mL tube using the hematoxylin and eosin-stained slide as a guide. Total RNA was extracted using the RecoverAll Total Nucleic Acid Isolation Kit (Ambion, Austin, TX). All RNA extractions were assessed for the amount and purity of RNA with Qubit 3.0 (ThermoFischer Scientific, Waltham, MA) and stored at -80 °C until further use. To avoid any potential variation among assays, analyses were performed on all the extracts simultaneously.

### Quantitative reverse transcriptase-polymerase chain reaction

PDCD4 reverse transcription was performed using 100 ng of total RNA, M-MLV Reverse Transcriptase (ThermoFischer Scientific, Waltham, MA), and 250 mM of random primers (ThermoFischer Scientific, Waltham, MA). PDCD4 primers (forward: 5’-TGGAAAGCGTAAAGATAGTGTGTG-3’; reverse: 5’-TTCTTTCAGCAGCATATCAATCTC-3’) were designed using the Probe-Finder software (roche-appliedscience.com) and the respective probe was selected among the Universal Probe-Library (Roche Diagnostics, Mannheim, Germany). Experiments were then performed according to the standard protocol provided by the manufacturer, including glyceraldehyde 3-phosphate dehydrogenase (GAPDH) as housekeeping gene control (primers sequences: AGCCACATCGCTCAGACAC forward and GCCCAATACGACCAAATCC reverse) to normalize the unequal RNA amounts. Mature miR-21 (primer sequence: 5’-GATACCAAAATGTCAGACAGCC-3’) was retro-transcribed from 100 ng of total RNA with the SuperScript VILO cDNA Synthesis Kit (ThermoFischer Scientific, Waltham, MA) and quantified using the NCode miRNA quantitative reverse transcriptase-polymerase chain reaction (qRT-PCR) method (ThermoFischer Scientific, Waltham, MA), according to the manufacturer’s instructions [36]. miRNA Cycle Threshold (Ct) values were normalized to small nuclear RNA U6B (primer sequence: 5’-GTCAGACAGCC-3’). All the reactions were run in triplicate, including no-template controls, on the Light- Cycler 480 Real-Time PCR System (Roche Diagnostics).

### Statistical analysis

Statistical significance was determined for IHC and qRT-PCR data by Mann–Whitney Wilcoxon test, comparing MPM and NNP cohorts and the MPM histopathological subtypes. Due to the low number of sarcomatoid MPM cases, biphasic and sarcomatoid MPM cases were considered jointly and compared to the epithelioid MPM cases. Differences between MPM and NNP cohorts in both miR-21 and PDCD4 qRT-PCR data were evaluated in terms of delta-Ct (ΔCt = Ct_MPM_ – Ct_NNP_) values. Prognostic relevance of PDCD4 was assessed using a publicly available microarray gene expression dataset from de Reyniès et al. [18] (pre-processed data from ArrayExpress with accession number: E-MTAB-1719); 20 MPM epithelioid samples were considered for overall survival analysis: 10 with low (i.e. <40^th^ percentile) and 10 with high (i.e. >60^th^ percentile) PDCD4 expression, considering the averaged signal across the corresponding probes. Patients were followed for a maximum of a 5-years overall survival. Log-rank test was performed comparing the Kaplan-Meier curves between the two sample groups defined above. All the analyses were performed using R statistical computing software (http://www.r-project.org), considering *p* values < 0.05 as statistically significant.

## CONCLUSIONS

The proposed explorative study suggests that PDCD4 could be a reliable and helpful immunohistochemical marker for MPM. PDCD4 diagnostic performances, as well as its prognostic relevance, should be tested in large MPM cohorts using both histological and cytological specimens. Functional experiments on PDCD4 and miR-21 pathway are needed to confirm our findings and to further investigate the molecular background underlying MPM oncogenesis.

## Abbreviations

MPM: Malignant Pleura Mesothelioma
PDCD4: Programmed cell death 4
IHC: immunohistochemistry
NNP: non-neoplastic pleura
ISH: *In-situ* Hybridization
CDKN2A: complex cyclin-dependent kinase inhibitor 2A
ARF: alternative reading frame
NF2: neurofibromatosis type 2
BAP1: BRCA1-associated protein
eIF4A: eukaryotic initiation factor 4A
eIF4G: eukaryotic initiation factor 4G
FFPE: formalin-fixed, paraffin-embedded
GAPDH: glyceraldehyde 3-phosphate dehydrogenase
qRT-PCR: quantitative reverse transcriptase-polymerase chain reaction
Ct: Cycle Threshold
EMT: epithelial-mesenchymal transition
FISH: Fluorescence *in situ* hybridization
